# Inability to walk due to scurvy: a forgotten disease

**DOI:** 10.4103/0256-4947.65266

**Published:** 2010

**Authors:** Hussein A. Algahtani, Abduljaleel P. Abdu, Imad M. Khojah, Ali M. Al-Khathaami

**Affiliations:** a Neurology Section, King Abdulaziz Medical City, Jeddah, Saudi Arabia; b College of Medicine, King Abdulaziz University, Jeddah, Saudi Arabia; c Neurology Section, King Abdulaziz Medical City, Riyadh, Saudi Arabia

## Abstract

Scurvy has become rare in modern societies, but should be considered in malnourished persons, alcoholics, and in infants on unsupplemented milk diets who present with musculoskeletal pain or a bleeding tendency. The diagnosis of scurvy can be challenging because of the rare incidence and vague and nonspecific early symptoms. We report here a case of scurvy in a young boy who presented with an inability to walk and severe musculoskeletal pain. The diagnosis was established based on his clinical picture, radiological appearance, and low serum level of vitamin C. The patient responded well to vitamin C supplementation with full resolution of his symptoms. He regained his ability to walk and his family was happy and satisfied with the outcome of treatment. Although the incidence of scurvy has become low in Saudi Arabia, it can still occur and early recognition is important because of the excellent prognosis.

Scurvy is a rare nutritional disorder that has been described for at least 500 years.^1^ It is caused by a prolonged deficiency of vitamin C intake that results in several metabolic abnormalities, including defective collagen synthesis and impaired tissue repair. Although rare in modern societies, scurvy should be considered in malnourished persons, alcoholics, and infants on unsupplemented milk diets.^2^

## CASE

A 6-year-old boy was brought to the neurology clinic with a 6-month history of inability to walk. Onset was gradual and the problem progressed slowly until the patient was bedridden. The boy was a product of a full-term uneventful pregnancy who was delivered through spontaneous vaginal delivery. There was no history of trauma, fever, weight loss or any developmental delay during infancy. Dietary history was significant in that he was drinking only milk during the last 3 years. His past medical and surgical history was unremarkable. He was conscious but irritable and resisted examination, lying on the couch with his legs flexed at hips and knees (Figure 1a). There were no rashes, petechiae, hematoma, or gingival bleeding. During the neurological examination, power in the lower limbs could not be assessed as he refused to exert at all. Reflexes were found to be exaggerated and plantars were bilaterally flexors. Upper limbs were normal. Cardiovascular examination was normal and there was no organomegaly. Respiratory system examination was normal, but examination of the chest cage revealed pectus excavatum with scorbutic rosaries (Figure 1b).

**Figure 1a:**
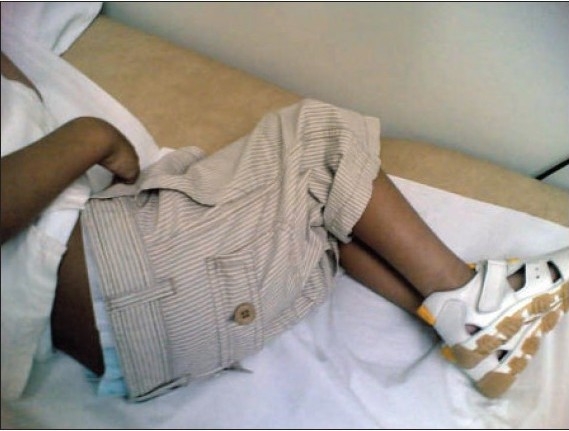
The child in flexion posture.

**Figure 1b:**
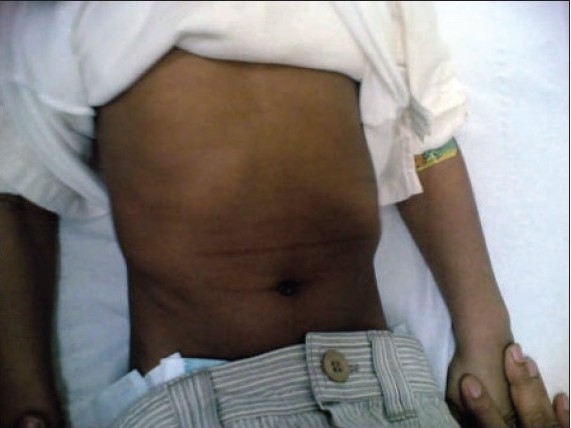
Photo of the chest cage with pectus excavatum and scorbutic rosaries.

X-ray of the lower limbs, upper limbs and pelvis showed the classical appearance of scurvy with a thin cortex and scurvy line. A vitamin C assay showed a markedly low level of 0.5 mg/L (normal, 4-20 mg/L). Other investigations, including a complete blood count, liver function tests, renal profile, calcium profile, thyroid function tests and vitamin D level were normal. CT of the brain was normal. The patient was started on a vitamin C supplement, 250 mg twice daily with a vitamin C rich diet. His condition improved dramatically and pain subsided. He started to move his legs and mobilized. Within 10 days he was back on his legs and walking with no support. Vitamin C supplementation was continued for 3 months and a follow-up vitamin C level showed that it was back to normal. The parents were educated regarding proper diet, and they started to follow a healthy well-balanced diet.

## DISCUSSION

Ascorbic acid is a water soluble, enoleic form of an alpha-keto lactone, a necessary cofactor in collagen biosynthesis.^3^ Normal collagen synthesis depends upon the hydroxylation of proline and lysine residues in the endoplasmic reticulum to form hydroxyproline and hydroxylysine, respectively. Failure of this step in the collagen synthesis results in impaired wound healing, deficient osteoblast and fibroblast function and defective tooth formation. It is also a reversible biologic reductant and provides reducing equivalents for a number of biochemical reactions involving iron and copper.^4^ This property enables ascorbic acid to function as a cofactor, enzyme complement, cosubstrate and strong antioxidant in a variety of reactions and metabolic processes. The antioxidant capabilities stabilize a number of other compounds, including vitamin E and folic acid. It is needed in the synthesis of dopamine, norepinephrine, epinephrine and carnitine. It has a role in prostaglandin and prostacyclin metabolism and hence it may be capable of attenuating the inflammatory response.^5^

Vitamin C (L-ascorbic acid) is absorbed in the small intestine through an energy-dependant process. It has a biological half-life of approximately 30 minutes. Humans are unable to synthesize ascorbic acid and therefore require an exogenous source for daily metabolic requirements.^6^ The daily requirement is obtained from natural sources such as citrus fruits and vegetables. The usual dietary doses (up to 100 mg/day) are almost completely absorbed. As dietary concentrations increase, a smaller fraction is absorbed. There is no storage site in the body, but some tissues carry higher concentrations (pituitary gland, adrenal glands, white blood cells and the eyes) and the total pool in the body is 1500-2500 mg.^7^ Ascorbic acid can be easily destroyed by heat, and therefore many foods can lose their ascorbic acid content because of cooking, storage or oxidation.

Scurvy is a clinical syndrome characterised by hemorrhage, hyperkeratosis, hypochondriasis and hematological abnormalities, but the presentation can vary by individual.^2^ Scurvy has been known to exist for more than three millennia, with description of similar conditions recorded by the ancient Egyptians in the Ebers Papyrus.^8^ It was a major cause of morbidity and mortality in much of Europe during the great potato famine and United States civil war. Captain James Cook was one of the first to demonstrate that sailors who spent months at sea could avoid scurvy by maintaining a diet rich in vegetables.^9^ In 1753, scurvy and its prevention by citrus fruits was systematically described by surgeon James Lind.

Clinical manifestations can be seen within 8 to 12 weeks of irregular or inadequate intake or when the body pool falls below 350 mg. Early clinical manifestations include pallor, myalgia, bone pain, irritability, poor weight gain, easy bruising, petechiae, perifollicular haemarroges, cork screw hairs, gum disease, poor wound healing, mood changes and depression. In advanced stages of scurvy, the patient is miserable and tends to remain in a characteristic immobilized posture from subperiosteal pain, with flexion of hips and knees. Late manifestations include genaralised edema, severe jaundice, fever, seizures, acute spontaneous bleeding and death. In infancy and childhood, the sternum may sink inward leaving a sharp elevation at the rib margins (scorbutic rosary).^10^

The incidence of scurvy is extremely rare in industrialized countries due to technological developments including food processing and transportation. It is still present in certain populations with poor nutrition including elderly persons living alone, those living in poverty, institutionalized or chronically ill patients, alcoholics and psychiatric patients. Scurvy is less frequent in the pediatric population.^11^ Infants who are fed with evaporated or boiled milk and children with a poor diet or those with psychiatric or developmental disorders are at risk. In our patient scurvy resulted from lack of vitamin C rich diet.

**Figure 2:**
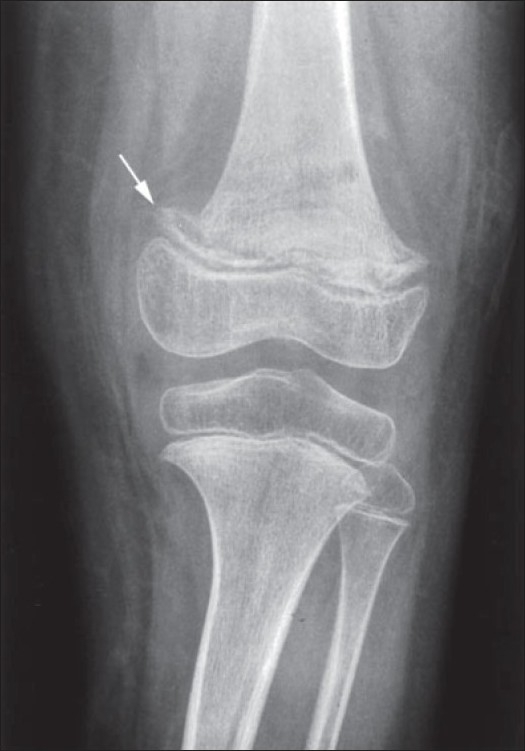
X-ray of the lower and upper limbs (arrow indicates scurvy line).

Skeletal X-ray changes are most evident at the end of long bones, particularly at the knee (Figure 2). These include atrophy and disappearance of trabeculae causing a “ground glass” roentgenographic appearance. The cortex is thin and an irregular white line appears at the mataphysis (Fraenkel line or scurvy line). Slipped diaphyses may be seen. Subperiosteal hemorrhage is common and during healing the elevated periosteum becomes highly calcified. Other changes include metaphyseal excrescences of the beaks, sub-epiphyseal infractions, increased density of periostitis and epiphyseal shell with a central lucency (Wimberger’s sign of scurvy).^12^ In our patient most of these classical features were seen. Because of the extremely rare occurrence of scurvy in modern society, the MRI findings of scurvy are not well described. These include subperiosteal hematoma with periostitis, metaphyseal changes and heterogenous bone marrow signal intensity.

A low plasma level of vitamin C is diagnostic of scurvy, which was seen in our patient. Measuring the vitamin C level in the Buffy-coat of the leucocytes better reflects the body stores. However, this method is technically more difficult.^13^

Normochromic, normocytic anaemia is common in scurvy and is due to bleeding into tissues. Macrocytic anaemia may also occur because of folate deficiency. This is due to the fact that many foods that contain vitamin C also contain folate and hence a diet that causes scurvy may also cause folate deficiency. Ascorbic acid deficiency also results in an increased oxidation of formyl tetrahydrofolic acid to inactive folate metabolites and may cause a decrease in the active folate pool.^10^ The anemia is corrected with supplementation of vitamin C and institution of a balanced diet.

The best evidence to confirm the diagnosis of scurvy is the clinical response to vitamin C supplementation with resolution of the manifestation of the disease within a few days, as occurred in this patient. Spontaneous bleeding usually ceases within 24 hours, muscle and bone pains subside quickly and the gums begin to heal within 2 to 3 days. Even large ecchymoses and hematomas resolve in 10 to 12 days. Complete recovery usually occurs after 3 months of adequate supplementation. In adults, the usual dose is 1 to 2 g of vitamin C administered daily for 2 to 3 days, followed by 500 mg per day for 2 weeks. Afterwards, 100 mg of vitamin C should be given daily for 1 to 3 months. In children, ascorbic acid 100 to 300 mg is given orally daily for 1 to 2 weeks, administered simultaneously with a nutritious diet supplying 1 to 2 times the recommended intake.^14^

Since scurvy is a potentially fatal condition, blood should be obtained for diagnosis and ascorbic acid treatment instituted promptly once suspected. Scurvy is potentially fatal if left untreated. The diagnosis can be missed easily as the disease presents with vague symptoms in the initial stages and cases are relatively rare, especially in modern societies. The disease still occurs in malnourished and those with peculiar diets. The importance of early diagnosis lies in the fact that it is an easily treatable condition with excellent outcome.
